# An Effective Delay Reduction Approach through a Portion of Nodes with a Larger Duty Cycle for Industrial WSNs

**DOI:** 10.3390/s18051535

**Published:** 2018-05-12

**Authors:** Minrui Wu, Yanhui Wu, Chuyao Liu, Zhiping Cai, Neal N. Xiong, Anfeng Liu, Ming Ma

**Affiliations:** 1 School of Information Science and Engineering, Central South University, Changsha 410083, China; mywmr@163.com; 2 College of computer and information engineering, Hunan University of Commerce, Changsha 410205, China; csuwyh@163.com; 3 School of Software, Central South University, Changsha 410075, China; liuchuyao@csu.edu.cn; 4 College of Computer, National University of Defense Technology, Changsha 410073, China; zpcai@nudt.edu.cn; 5 Department of Mathematics and Computer Science, Northeastern State University, Tahlequah, OK 74464, USA; xiongnaixue@gmail.com; 6 The State Key Laboratory of Industrial Control Technology, Zhejiang University, Hangzhou 310027, China; 7 Department of Computer Science, Stony Brook University, Stony Brook, NY 11794, USA; minma@cs.stonybrook.edu

**Keywords:** industrial wireless sensor networks, duty cycle, sleep delay, energy efficient, lifetime

## Abstract

For Industrial Wireless Sensor Networks (IWSNs), sending data with timely style to the stink (or control center, CC) that is monitored by sensor nodes is a challenging issue. However, in order to save energy, wireless sensor networks based on a duty cycle are widely used in the industrial field, which can bring great delay to data transmission. We observe that if the duty cycle of a small number of nodes in the network is set to 1, the sleep delay caused by the duty cycle can be effectively reduced. Thus, in this paper, a novel Portion of Nodes with Larger Duty Cycle (PNLDC) scheme is proposed to reduce delay and optimize energy efficiency for IWSNs. In the PNLDC scheme, a portion of nodes are selected to set their duty cycle to 1, and the proportion of nodes with the duty cycle of 1 is determined according to the energy abundance of the area in which the node is located. The more the residual energy in the region, the greater the proportion of the selected nodes. Because there are a certain proportion of nodes with the duty cycle of 1 in the network, the PNLDC scheme can effectively reduce delay in IWSNs. The performance analysis and experimental results show that the proposed scheme significantly reduces the delay for forwarding data by 8.9~26.4% and delay for detection by 2.1~24.6% without reducing the network lifetime when compared with the fixed duty cycle method. Meanwhile, compared with the dynamic duty cycle strategy, the proposed scheme has certain advantages in terms of energy utilization and delay reduction.

## 1. Introduction

With the rapid development of microprocessor technology and embedded systems, the number, function, and handling capacity of sensing devices has rapidly developed [1,2]. A significant number of sensing devices is being applied to various applications in a large scale [3,4,5,6], such as intelligent transportation systems [7,8] and smart grids or smart health [9,10], among others. These capabilities can also be extended into production, manufacturing, logistics, and maintenance areas, in which communication capabilities combined with data analysis and advances in Cyber Physical Systems (CPS) provide the grounds for the advent of industry 4.0 environments [11,12,13,14]. Reports from Cisco show that the size of various embedded perceptual devices connected to the network is now unprecedented [15,16]. Since 2011, devices connected to the Internet of things on the earth (such as industrial sensing devices and smart phones, etc.) have exceeded the human population, reaching nine billion devices. It is expected that by 2020, 24 billion sensing devices will be connected to the network [15,16]. This large number of sensing devices is linked to the network, forming a new network structure and computing model called the Internet of Things (IoTs) [2,8,13,15,16,17,18], or the Edge network [19]. The sensor network and cloud network combine to form the Sensor-Cloud Network (SCN) [20,21,22]. In SCN, the deployed sensor nodes are no longer just for specific applications, but are unified by the cloud to accept application requests. Then, the cloud releases the requirements of the application to the sensor nodes for the corresponding perception and data collection. The sensor nodes then accept the cloud command and operate the site accordingly. Thus, the application, scope, and depth of sensor networks are significantly augmented.

Industrial Wireless Sensor Networks (IWSNs) are an important application field of Wireless Sensor Networks [13,14]. The trend in the field of industrial automation is the use of wireless technology and wireless control applications [23]. In all kinds of industrial applications, wireless sensor nodes are widely used because of their small size, flexible deployment, low requirements for deployment in an environment, and wireless automatic networking [24,25,26,27,28]. WirelessHART, the first open wireless communication standard, which is specially designed for applications in industrial automation, was released in September 2007. Unlike general sensor networks, in the WirelessHART network, normal sensors are connected to actual devices to collect specific environmental data [29]. For example, in an industrial production line, wireless sensor nodes can be deployed flexibly and change with the production line, allowing for the accurate collection of images and sounds during production. As a result, the mechanical conditions in the production line can be monitored in real time. Once an abnormal situation is monitored, it is quickly reported to the control center and corresponding control measures to ensure the efficiency and safety of production are initiated to avoid loss of property and personnel [13,14]. However, an industrial wireless sensor network is relatively sensitive to communication delay when compared to other applications. If an emergency is not handled in a timely manner, this may result in significant loss of personnel and property [13,14,18,23,30,31,32,33]. For example, in the monitoring of an industrial smelting furnace, when the temperature of the smelting furnace reaches a certain value it must be treated in time. If the temperature is too high and not processed in time, it will cause the explosion of the smelting furnace and the loss of personnel and property. If the temperature is too low, it will solidify the metal liquid of the smelting furnace and damage the smelting furnace and cause property loss [13,14]. While in the industrial setting, these critical values last for a short time and must be processed in a timely manner. Therefore, in the design and implementation of IWSNs, how to reduce a data routing delay is a critical issue [30,31]. 

Researchers define the total time required for data to be transmitted from the source node to the sink as end to end delay [13,14,23,30,31]. In the process of routing [32,33,34,35], the time that data is received from one node to be successfully transferred to the next hop is a single hop delay. Because end to end delay is the accumulation of the single hop delay for each node in the routing process [18], the farther the distance from the source node to the sink, the more hops on the routing path, and consequently the greater the end to end delay. Thus, reducing the single hop delay is a fundamental and effective way to reduce the end to end delay. But the single hop delay (or delay for short) is affected by many factors [18]. After receiving the data packet, the node first requires a certain processing time to receive and process it from the receiving port before finally entering it into the sending queue. Then, it transfers to the next hop according to the transport protocol until the next hop node successfully receives the packet [36,37,38,39,40]. Thus, the delay includes the processing time of the data, the queuing time, and the sending time. The data may be sent many times and are dependent on the transport protocol. Among them, the time of node processing data is very small, and it can be neglected in the wireless transmission environment. The queuing time is determined by the load of nodes. The more data the network needs to transmit, the longer the queuing time of the packets, and transmission will stop in the event the queuing columns are full [13,14,30,31]. The sending time of data is mainly influenced by the data transport protocol and many other network factors. The sending time under different data transport protocols is different. For example, in the send-wait protocol, when the sender sends a packet, it will wait for the receiver to return the ACK message. If the sender receives the ACK message, it can be confirmed that the packet has successfully reached the receiver [41]. On the contrary, the sender will re-send the data packet after waiting for the timeout until the packet is sent successfully, or the number of retransmission times exceeds the prescribed number and then the transmission of the packet will be abandoned. The time required for different data transmission is different, and the network conditions also have a great impact on the delay under the same data transport protocol. If the success rate of the packet transmission is high, the probability of the data retransmission is small and the delay is small. Therefore, network conditions affect the probability of successful data transmission. If the sender uses greater transmission power, this can improve the signal-to-noise ratio and shorten the time required for data transmission, ensuring that the success rate of data transmission is high [41]. 

Under the circumstance that the network conditions and data transmission protocols have been determined, one of the most important factors that can affect delay is the duty cycle mechanism used by IWSNs. In Wireless sensor networks (WSNs), as the energy of the node is very limited, the extension of lifetime is a key factor in network design to effectively reduce energy consumption. The duty cycle mechanism is an effective way to reduce the energy consumption of sensor nodes [42]. The principle of this method is: the energy consumption of a sensor node in the awake state is more than 100 times of its energy consumption in the sleep state. In order to reduce the energy consumption of the nodes, the nodes must sleep when they do not need to work. As a result, the duty cycle mechanism means a periodic transformation of nodes between awake and sleep states to reduce energy consumption. Generally speaking, the cycle frequency of the duty cycle is relatively high, while the cycle time of physical events is very large relative to the duty cycle. Therefore, sensor nodes can make use of the duty cycle mechanism to save energy when meeting the monitoring requirements. However, the node cannot receive and send data when it is in the sleep state. Therefore, when the sender needs to transmit data, if the nodes in the forwarding nodes set are all in the sleep state, then the sender needs to wait for its forwarding nodes to wake up before beginning data transmission [42]. In this paper, the time that the sender node must wait for its forwarding nodes due to the duty cycle mechanism is called sleep delay. The sleep delay depends on the duty cycle of the sensor nodes. In a period, the ratio of the time of the node in the awake state and the whole cycle time is called the duty cycle (denoted as δ). In some environmental monitoring applications that are not strict in real time requirements, the cycle of the duty cycle may be measured in minutes when the temperature is collected [43,44]. Therefore, in this case, sleep delay is the leading factor in end to end delay. Even in IWSNs, the sleep delay of sensor nodes has a great effect on the end to end delay [42]. Thus, how to reduce the sleep delay in data routing is a key factor in reducing end to end delay. Another delay associated with the duty cycle is the delay for detection. When a node is in the sleep state it cannot detect data packets. When a packet is sent to the node in the sleep state it is necessary to wait for the node to wake up before the packet can be perceived and received. The elapsed time between the node sending the packet and the other node perceiving the packet is called the delay for detection.

The reduction of sleep delay and delay for detection are also major challenges. Because delay is caused by the duty cycle mechanism used by the sensor nodes [42], it essentially depends on the time that the node is in the sleep state. To reduce the sleep delay it is necessary to reduce the time of the node in the sleep state and increase the time that it is awake. In other words, this increases the duty cycle δ. Increasing the duty cycle will increase the energy consumption of the node and reduce its lifetime. Because of this, as far as we know, there has been no research into maintaining a high lifetime while reducing delay.

In this paper, we provide an optimization method that can effectively reduce the delay and ensure that the lifetime is not less than the previous strategies. Specifically, the main innovations in this work are as follows:

(1) A novel Portion of Nodes with Larger Duty Cycle (PNLDC) scheme is proposed to reduce sleep delay and maintain a high lifetime for IWSNs. We have taken note of the following two facts: First, the sender has multiple forwarding nodes and the sleep delay is equal to 0 as long as the duty cycle of any node in the forwarding nodes set (FNS) is 1. Second, a significant number of studies show that the nodes in the near sink region bear a lot of data so their energy consumption is high while the nodes far away from the sink have residual energy. Therefore, a PNLDC scheme makes full use of the residual energy of non-hotspots to set the duty cycle of a certain proportion of nodes to 1 (δ=1). Thus, a PNLDC scheme is able to reduce delay while ensuring that lifetime is not less than the previous strategy.
(2) Through strict theoretical calculation and deduction, this paper first gives the proportion of nodes with full duty cycle (duty cycle = 1) in different regions that have different distances to the sink. Then, the relationship between the proportion of nodes with full duty cycle and the reduced delay is given. This provides the theoretical basis for the PNLDC strategy and provides the basis for the calculation of similar methods.
(3) The full theoretical analysis in this paper shows that the proposed PNLDC scheme significantly reduces the delay for forwarding data by 8.9~26.4% and delay for detection by 2.1~24.6% without reducing the network lifetime when compared to the fixed duty cycle approach.

The rest of this paper is organized as follows. In Section 2, the related works are reviewed. The system model and problem statements are described in Section 3. The PNLDC scheme is proposed in Section 4. The performance analysis and simulation results of PNLDC scheme are provided in Section 5. Finally, we conclude in Section 6.

## 2. Related Work

Industrial wireless technology is a revolutionary technology that can reduce the cost of industrial measurement, its control systems, and improve upon the application scope of the system. At present, three international standards have been formed in the field of industrial wireless technology, namely, the WirelessHART standard, the ISA100.11a standard, and the WIA standard. Song et al. first published a report on building the WirelessHART protocol stack [29]. In their research, the WirelessHART standard and its overall architecture was introduced, and several challenges that must be addressed in the prototype implementation were described and solved, including reliable mesh network, communication security, whole network synchronization, and timer design. So far, many studies have proposed improvement protocols for reliability and real-time assurance [45,46,47,48,49]. Johan et al. summarized future research challenges of wireless sensor networks applied to industrial automation and pointed out that safety, reliability, and availability are the main issues to be solved [23]. Among them, safety is the most important feature that has been considered so far, while other important features include the interoperability, availability, and assurance of real-time performance, in which delay is an important parameter that requires specific attention.

The end to end delay is larger because of the easy loss of data transmission in wireless sensor networks, multi hop transmission, and wireless conflict [50,51,52,53,54]. In order to reduce data transmission delay, researchers have put forward many effective methods [13,14,18,24,30,31,41]. These methods are summed up in the following categories in this paper.

(1) The delay minimization at the network routing level. This method of delay optimization on the network routing level is one of the most common research areas for researchers [55,56]. A variety of routing methods have been proposed. The shortest routing algorithm was first proposed. The principle of this method is to reduce delay. Because the delay in the arrival of the packet to the sink is the sum of the delay for each hop in the routing process, reducing the number of hops on the routing path can reduce the end to end delay accordingly. The most effective way to reduce the number of hops in the routing path is to choose the shortest path from source node to sink for data transmission. At this time, the number of hops is the least and so the delay is also the minimum [55]. 

However, choosing the shortest route does not always reduce the delay. In some cases, avoiding congested areas will increase the route length, but can oftentimes reduce the delay. In some cases, the transmission path will often bypass energy consumption hotspots to avoid energy consumption of nodes in this area that are too fast to die [56]. Thus, the method of reducing the length of the transmission path to reduce the delay is limited by some restrictions. And in many cases, the effect of the path length on the delay is far less than the other factors. Therefore, some quality of service (QoS) based routing methods are then proposed [42]. These QoS methods mainly include data transmission reliability, security, transmission rate, and many other indicators. Delay is only one of a variety of QoS indicators.

Because multiple QoS indicators are interrelated and interact with each other, the next hop selection of routing becomes a multi-objective optimization problem, which makes routing more complex. For example, the reliability of data transmission refers to the probability that a packet is transmitted to the sink successfully [14,36]. Obviously, if the high reliability is guaranteed, more data retransmission may be needed, thus increasing the delay [41]. In the multipath routing strategy, which guarantees data packets arriving to the sink safely, this strategy copies data packets into multiple copies, and each copy is transmitted to the sink through different paths. So, even if some transmission paths are attacked, there is still a path to successfully reach the sink, thus ensuring the security of the network. In such a routing strategy, delay is the time required to arrive at the sink first. Therefore, in this case, the delay can be effectively reduced. Therefore this way of routing is often used to improve the reliability of data transmission [56,57,58].

(2) The effect of the communication link quality and data transmission mechanism on delay [14,36]. Because of the unreliability of network transmission, data transmission is often not successful for the first time, and some reliability systems need to be adopted to ensure the reliability of data transmission. These reliable data transmission mechanisms have an important impact on the delay. The simplest data transmission mechanism is the send-wait mechanism. Under this mechanism, the sender sends data packets and waits for the receiver to return the ACK message to the sender. Once the sender receives the ACK, it indicates the receiver has successfully received the packet, then the sender starts sending the next packet [41]. Otherwise, the sender waits for a longer timeout time and resends the current packet. The above process goes on until the packet is sent successfully, or the number of retransmission times exceeds the predetermined threshold and then the transmission of this packet will be abandoned. It can be seen that in this way, the time of a packet retransmission is greater than round trip time (RTT). Therefore, in a network with poor communication link quality, its delay is often several times the delay in the ideal communication link [41]. The reason why the delay of a simple send-wait protocol is large is that the sender sends a packet and must wait for the receiver to return the confirmation information. If the packet is a loss, the sender needs to wait for a longer timeout time, which results in greater delay. The main idea of the related improved method is to send multiple data packets continuously while the receiver also continuously returns the confirmation information, then the sender reissues the packets that did not have confirmation information [41]. It can be said that this method uses a parallel working mechanism to reduce the delay. The protocols for this kind of improvement are: Go back-N (GBN) protocol and Selective repeat (SR) [41] algorithm.

(3) The effect of transmission power on delay [14,36]. It can be seen from the above that if the quality of the communication link is not good, in order to ensure a certain reliability of data transmission, a large maximum retransmission number of data must be set. However, in this case, not only is the delay very large, the energy consumption is larger. Therefore, improving the quality of communication link can effectively reduce delay and so on. The way to improve the communication quality is to improve the signal-to-noise ratio of the communication link. The higher the signal-to-noise ratio of the communication link is, the higher the probability of the correct data reception is, the smaller the number of data retransmission is, and the smaller the delay is. The signal-to-noise ratio of the communication link can be improved by increasing the transmission power of the sender, which can effectively reduce the delay. However, this method improves the energy consumption of data transmission, so researchers have provided a method to determine the transmission power of the optimized sender [14,36].

(4) The reduction of delay based on broadcast based routing. The common routing method is the way that a sender corresponds to a receiver. Thus, in this way, if the receiver does not receive the packets correctly, it is necessary to carry out the retransmission work described above, which leads to a larger delay. The opportunity routing method described below can effectively alter this situation [36].

Opportunistic routing [59] is based on the following method to reduce the delay on the basis of ensuring the reliability of data transmission. First, the sender determines a candidate set, which comes from the Forwarding Nodes Set (FNS). The forwarding nodes set is a collection of such nodes: the nodes are in the sending range of the sender node, but are closer to the sink than the sender. The candidate set contains the nodes in FNS that are close to the sink and have high reliability. When the sender transmits data, it sends the data to all the nodes in the candidate set using the broadcast transmission. As long as any node in the candidate set receives the data, the transmission is successful. Because data will be retransmitted only if multiple candidate nodes do not receive it and the probability that multiple candidate nodes do not receive data is low, the probability of retransmission is minimal. In other words, it can effectively reduce delay [59]. 

It can be seen that in the broadcast transmission method of low reliability network, one sender corresponds to multiple receivers. The retransmission is caused only when multiple receivers have not received the data packet. Therefore, it can effectively reduce the number of retransmission. This method has also been studied in Ref. [60]. Joo et al. [60] studied this mode of broadcast transmission for data fusion networks, which consists of N packets into a packet. In their research, the network adopts the broadcast way to carry on the data transmission. After broadcasting, the data is received by multiple receiving nodes. The packets received by these receiving nodes continue to broadcast transmission. Therefore, using this method, retransmission is rarely needed in a low reliability network, which reduces the delay on the premise of ensuring reliability.

The delay minimizing scheme described above is based on such a hypothesis: nodes in the network are always in the awake state. In this case, the receiver is always able to receive the signal when the sender has a packet to send. But in practice, in order to save energy, many sensor networks use the duty cycle mechanism [42,61]. In this mechanism, the node converts between two states of activity and sleep. As a result, when the sender has a packet to transmit, the nodes in the forwarding nodes set are not necessarily in the active state. Thus, the sender needs to wait for a period of time until the forwarding node wakes up, then the data can be transmitted. This sleep delay is often much larger than the time used for data transmission [42,61]. So, in the duty cycle based network, it is more important to reduce the sleep delay due to the duty cycle mechanism, in addition to the earlier methods mentioned above. 

There are also many factors affecting the sleep delay, in which the node density has a certain effect on the delay [42,61]. The greater the density of the nodes the smaller the sleep delay is when the duty ratio is fixed. This is because the greater the node density is, the larger the forwarding nodes set is when the sender sends data. The work pattern of the node is asynchronous, so the expected value of waking of the candidate forwarding nodes is smaller. For a wireless sensor network, once the nodes are deployed, the density of nodes is fixed. Therefore, another important factor affecting the sleep delay is the duty cycle [42,61]. If the duty ratio is large, for example, its value of 1 means that in one cycle the node is always in the active state without sleep. Therefore, the sleep delay is 0, which is the minimum. But the large duty cycle consumes more energy of the node, which seriously reduces the lifetime of the node. For example, the lifetime of a node with a duty cycle of 1 is only about 1/3 of the lifetime of a node with a duty cycle of 0.3. Therefore, it is not a good method to simply adopt the method of increasing the duty cycle. As a result, researchers are seeking to reduce the delay without changing the duty cycle. The improvements in routing algorithms are also conducive to reducing delays in wireless sensor networks based on the duty cycle. It is very different from the previous consideration of the number of hops on the transmission path. In the non-duty cycle network, the sender can select the nearest neighbor node from the sink to forward the data every time. There is a different situation in the network based on the duty cycle. When the sender has data to transmit, the nearest neighbor node from the sink in its forwarding nodes set is not necessarily in the active state. At this point, the sender has two options. One is to continue to wait for the nearest neighbor node from the sink in the forwarding nodes set until it wakes up. In this way, the distance of transmission is far each time, so the number of hops needed for the data transmission to the sink is less, but each hop takes a long time to wait. The two are not waiting, but are transferring the data to a neighbor node that is in the active state and is closer to the sink than the sender. Although the distance of one hop in the transmission is not very large, more hops are needed in the transmission path to the sink. But compared to the first choice, the delay of each hop is smaller. It can be seen how to select the appropriate forwarders is a very complex problem. From another point of view, Naveen et al. solved the problem of the forwarding node selection in the delay minimization [42]. They abstracted the selection of forwarding nodes in the sleep-wake cycle WSNs into the Asset Selling Problem (ASP) [42], and proved that this is a NP complete problem.

It can be seen that it is a challenge issue to reduce delay in duty cycle based WSNs. A novel solution is proposed in this paper from a new point of view. We notice that when the sender sends data, there are multiple candidate forwarding nodes, therefore only one node in these nodes wakes up so that data can be forwarded. This inspires the following idea: if the duty cycle of a part of the forwarding nodes is set to 1, then they will always be in active state. Although the energy consumption of the node with a duty ratio of 1 is large, the duty cycle of most nodes is still smaller because of the small proportion of the nodes set to 1. Therefore, the energy consumption of the network is not high and it can effectively reduce the sleep delay. More importantly, we found that in WSNs the energy consumption in the area near the sink is high and the energy consumption of the nodes far away from the sink is small. According to the related research, the area far away from the sink left eighty percent of the network energy. Therefore, we make full use of the energy of the nodes far away from the sink to select a certain proportion of nodes and set the duty cycle of them to 1. It is not fixed that the duty cycle of some nodes is always 1. Thus, this method makes full use of the network energy, balances the energy consumption of the network, effectively reducing the delay, and does not affect the life of the network, which is very important for the resource strained network [62,63,64]. The method proposed in this paper is also innovative.

## 3. System Model and Problem Statements

### 3.1. System Model

In this paper, the system model is a typical wireless sensor network based on duty-cycled, which is similar to the model in Ref. [61]. There are n sensor nodes randomly deployed in a two-dimensional network. The radius of this network is *R* and the node density is ρ. And the communication range of each node can be seen as a circular area with a radius of *r*, with the sender node as the center. Therefore, in the process of transferring from the initial sender to the sink, the packet may pass through multiple nodes. This means that each node can be either a receiver or a sender. According to the communication range and corresponding method of selecting relay nodes, the candidate forwarding nodes for each node can be determined. Since the energy of the node is limited, the method of duty-cycling based on the asynchronous protocol is used here. In this method, the state of nodes converts between two modes, namely, sleep modes and active modes. Each node wakes up at different times. When a sender node has a packet to transmit, if its candidate forwarding nodes are not in active mode, it must wait until at least one of its forwarders wakes up.

In the entire network, packets or events are randomly distributed. Therefore, the probability of each sensor node’s perception of a packet is equal. Likewise, for each node, the probability that it generates data is equal.

### 3.2. System Parameters

Sensor nodes are mainly composed of two units: a communication unit and sensing unit. Among them, the sensing unit is in charge of the perception of network events and invoking the communication unit for packet transmission. Because in the network sensor nodes can both perceive and forward packets, the energy consumption of nodes is serious. To reduce the energy consumption in the system, as mentioned above, this network uses a method called duty-cycling so that the sensor nodes can make a periodic conversion between active modes and sleep modes according to the specified wake-up interval. Therefore, the time is divided into some frames of the same length in the working cycle, and each frame is composed of an active state and a sleep state. In the active state, nodes can both send and receive packets. In contrast, nodes cannot complete these operations in the sleep state, which saves energy. Based on the above description, in a unit period, the duty cycle of each node can be defined as the ratio of the time of the node in the active mode and the time of the entire unit cycle. 

In this paper, for sensor nodes, there are three main periods: communication, sensing, and self learning. In the self learning period, nodes remain in an active state and perceive the state of their candidate forwarding nodes. After this period, the sender nodes start to communicate. The communication duty cycle is indicated by $\delta_{COM}$, and the sensing duty cycle is denoted as $\delta_{SEN}$. $\delta_{COM}$ and $\delta_{SEN}$ can be expressed by Equations (1) and (2).

(1) $$\delta_{COM}=T_{COM}^{A}/\left(T_{COM}^{A}+T_{COM}^{S}\right)$$

(2) $$\delta_{SEN}=T_{SEN}^{A}/T_{SEN}=T_{SEN}^{A}/\left(T_{SEN}^{A}+T_{SEN}^{S}\right)$$

where $T_{COM}^{A}$ is the time that nodes in the active mode during the communication period and $T_{COM}^{S}$ is the time that nodes in the sleep mode during the communication period. In the sensing duration (denoted as $T_{SEN}$), $T_{SEN}^{A}$ is the duration when nodes in active state and $T_{SEN}^{S}$ is the duration when nodes in sleep state.

In most of the previous research, the duty cycle of all sensor nodes is the same. But in this paper, we will select some nodes in the non-hot areas on the basis of self learning, and set the duty cycle of them to 1, so as to reduce delay, improve the quality of monitoring packets and effectively use energy. Thus, the power consumption of nodes mainly includes four parts: (1) the power required to send or receive packets, denoted as $\mu_{T}$ and $\mu_{R}$. (2) the power used in low power listening (*LPL*), denoted as $\mu_{LPL}$. (3) the power consumption of sensing when nodes in active state, denoted as $\mu_{SEN}$. (4) the power used when nodes in sleep state, denoted as $\mu_{S}$.

In this system model, the main parameters are listed in the Table 1 [42,61]. The other parameters are explained in the later text.

### 3.3. Problem Statements

In this paper, the main goal is to propose an approach to improve the network energy efficienciency and reduce the delay in transmission while maximizing the network lifetime. This goal can be characterized by the following indicators as explained below:

(1) The energy utilization (denoted as ${Κ}_{UTI}$) is defined as the ratio of the energy used by the entire network to the initial total energy. The improvement of network energy utilization refers to increasing the effective utilization of energy, that is, maximizing the ratio of energy consumption of the entire network to the initial total energy. The energy utilization can be calculated by the equation below.

(3) $$max\left({Κ}_{UTI}\right)=max\left(\frac{\sum_{i=1}^{n}\xi_{i}}{\sum_{i=1}^{n}E_{ini}}\right)$$

(2) The total delay (denoted as $Y_{TOT}$) in the network is related to the waiting time before the transmission of each hop and the time that the sender takes to transmit the packet to the sink. Therefore, the total delay can be divided into two parts: the delay for detection and the delay for forwarding data. The target of reducing the delay can be expressed as:

(4) $$min\left(Y_{TOT}^{k}\right)=min\left(\sum Y_{DET}+Y_{i,j}\right)$$

where $Y_{DET}$ represents the delay for detection and $Y_{i,j}$ represents the delay in forwarding data from node *i* to node *j*, node *j* is the forwarding node of node *i*, and nodes *i* and *j* are in the transmission path from the node *k* to the sink.

(3) The network lifetime (denoted as L) is defined as the time when the first node in the network dies out due to energy exhaustion. It means that the maximization of network lifetime is closely related to the maximum of energy consumption. The goal of maximizing the network lifetime can be expressed as follows.

(5) $$max\left(L\right)=min\left(max\left(E_{T}^{x}+E_{R}^{x}+E_{LPL}^{x}+E_{SEN}^{x}\right)\right)$$

In conclusion, the goal of the approach proposed in this paper can be expressed as:

(6) $$\{\begin{array}{c} max\left({Κ}_{UTI}\right)=max\left(\frac{\sum_{i=1}^{n}\xi_{i}}{\sum_{i=1}^{n}E_{ini}}\right)\\ min\left(Y_{TOT}^{k}\right)=min\left(\sum Y_{DET}+Y_{i,j}\right)\\ max\left(L\right)=min\left(max\left(E_{T}^{x}+E_{R}^{x}+E_{LPL}^{x}+E_{SEN}^{x}\right)\right) \end{array}$$

## 4. The Design of PNLDC Approach

### 4.1. Research Motivation

In wireless sensor networks, because each node has a transmission range, data packets usually need to pass through multiple hops on the transmission path. Thus, the farther the distance between the sink and the sender node, the more hops that the packet needs to pass through. At the same time, the network uses the duty-cycling method based on the asynchronous protocol, which means that the nodes make a periodic conversion between active modes and sleep modes. Therefore, packets can be sent and received only when corresponding nodes are in the active state. But for each hop on the transmission path, it does not guarantee that the receiver is in the active state. If the receiver is in the sleep state, the sender node must wait until it wakes up, and the worst case is that the waiting time is close to the whole cycle duration. As a result, the more hops on the transmission path, the larger the transmission delay. It can be obtained that the transmission delay of the nodes in the area far away from the sink is large, which will affect the communication delay of the entire network. Considering that the distribution of sensor nodes in the network is fixed, it is necessary to reasonably plan the working mode of nodes to reduce the transmission delay.

There is good deal of research on the effective reduction of transmission delay between nodes and their candidate forwarding nodes in duty-cycled wireless sensor networks. Most of the proposed methods adopt an optimal value of the duty cycle, that is, the duty cycle of all nodes in the network are the same. In these methods, some other network performance often needs to be sacrificed to reduce the delay. This is because these methods usually increase the time of the nodes in the active state of a work cycle, that is, increasing the duty cycle of nodes. But after improving the duty cycle, since the energy consumed when a node is in the active state is more than one thousand times of the energy consumed when it sleeps, the energy consumption of the network will be improved. Considering that the maximum energy consumption has a great impact on network lifetime, reducing the delay on the premise of guaranteeing energy consumption that does not affect the network lifetime is a problem that should be solved urgently.

**Figure 1:**
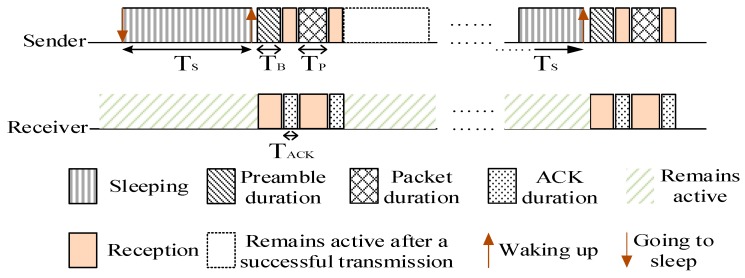
Timeline of nodes with basic duty cycle.

**Figure 2:**
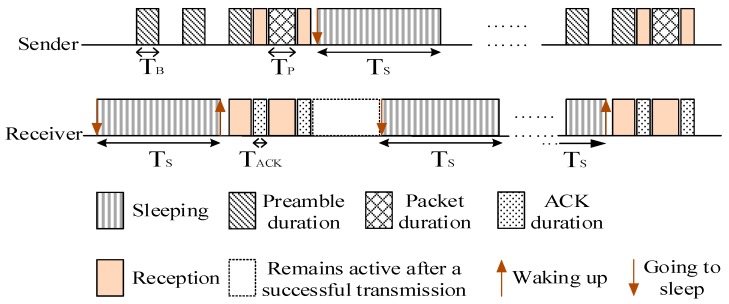
Timeline of nodes with full duty cycle.

Many studies show that the nodes in the region near the sink undertake a lot of data forwarding. Thus their energy consumption is high while the nodes far away from the sink maintain residual energy. Related studies indicate that the region far away from the sink held about eighty percent of the network energy. Selecting a proportion of nodes in the non-hotspots and setting their duty cycle to 1 can reduce the delay while using the residual energy. When there is at least one node with the duty cycle of 1 in the forwarding nodes set of the sender, the sender does not need to wait for the receiver to wake up before the transmission. This will effectively reduce delay. The comparison between the nodes with basic duty cycle and the nodes with the duty cycle of 1 is shown in Figure 1 and Figure 2.

**Figure 3:**
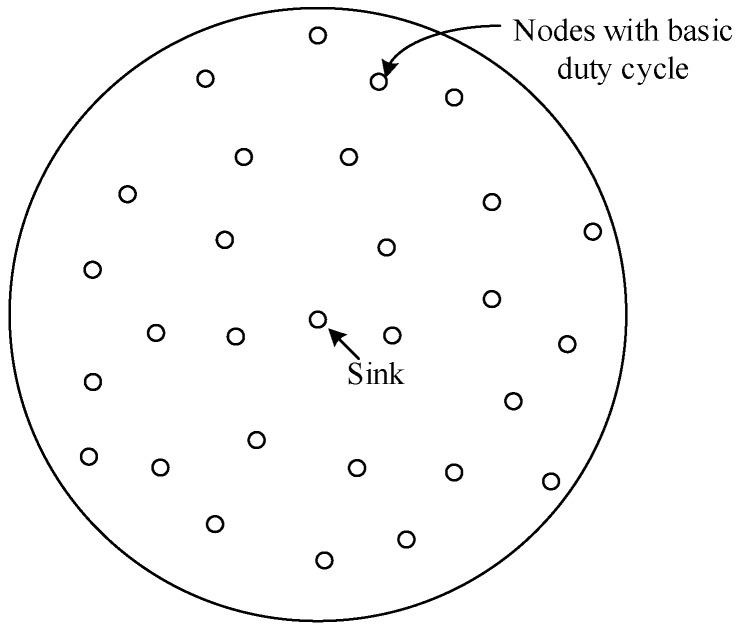
The distribution of all nodes with a basic duty cycle.

The distribution of the nodes corresponding to Figure 1 is shown in Figure 3. The white nodes represent nodes that adopt a basic duty cycle.

According to Theorem 1 in Ref. [61], considering that the length of a duty period is ρ and in a duty period the length of the time that node in the active state is $T^{A}$, then the duty cycle of each node is $\frac{T^{A}}{\rho }$. And the number of nodes in the forwarding nodes set is *p*. In this situation, the sender can forward the packet if there is a node in FNS in the active state, so the expected delay can be calculated by Equation (7).

(7) $$Y_{FFSC}=\sum_{i=0}^{\frac{\rho }{T^{A}}-2}i\left[1-{\left(1-\frac{T^{A}}{\rho }\right)}^{2}\right]{\left(1-\frac{T^{A}}{\rho }\right)}^{pi}+\left(\frac{\rho }{T^{A}}-1\right){\left(1-\frac{T^{A}}{\rho }\right)}^{p\left(\frac{\rho }{T^{A}}-1\right)}$$

**Figure 4:**
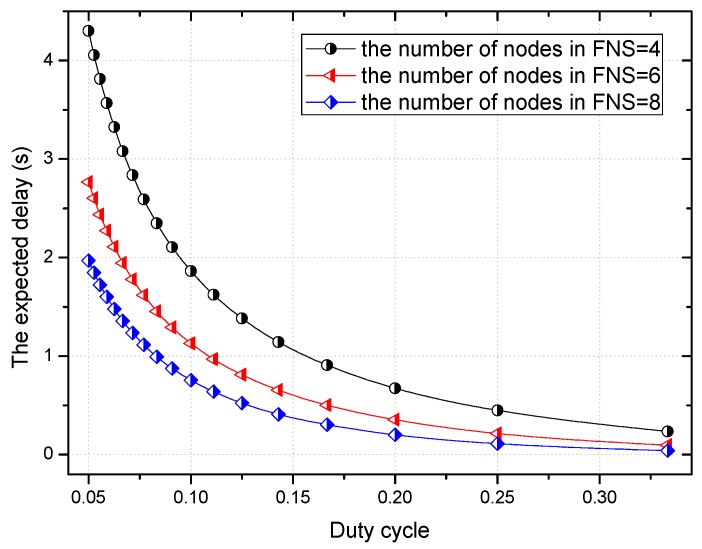
The expected delay decreases as the duty cycle increases.

Based on Equation (7), Figure 4 shows the relationship of the delay, and the number of nodes in the FNS and the duty cycle. With the increase of the duty cycle, the expected delay decreases. This is because a longer duty cycle means that the nodes are in the active state for a longer period of time. Therefore, the waiting time before the receiver wakes up is reduced. It can also be seen that as the number of nodes in FNS increases, the expected delay decreases. This is because each node in this network has an independent duty cycle, with the increase of the number of nodes in the FNS, the probability that a node in the FNS is exactly in the active state when the sender has a packet to send is greater.

**Figure 5:**
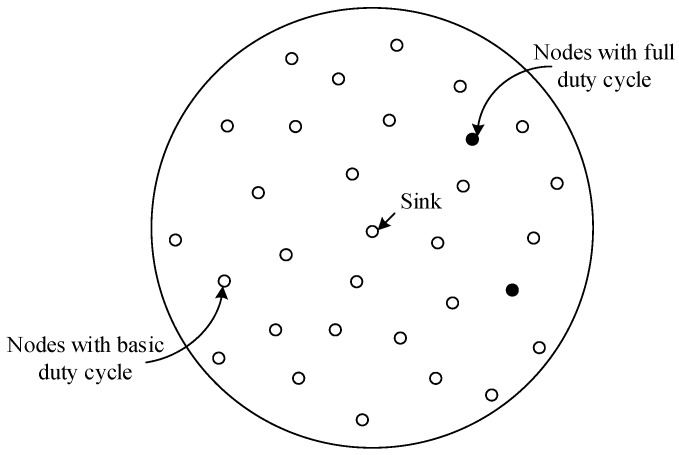
The distribution of two kinds of nodes.

The distribution of the nodes corresponding to Figure 2 is shown in Figure 5. The black nodes represent nodes that adopt a full duty cycle, that is, δ=1. And the white nodes represent nodes that adopt a basic duty cycle. In this situation, selecting some nodes and setting their duty cycle to 1 can reduce delay. This is because as long as a node in the forwarding nodes set is in the active state, data transmission can be carried out.

**Figure 6:**
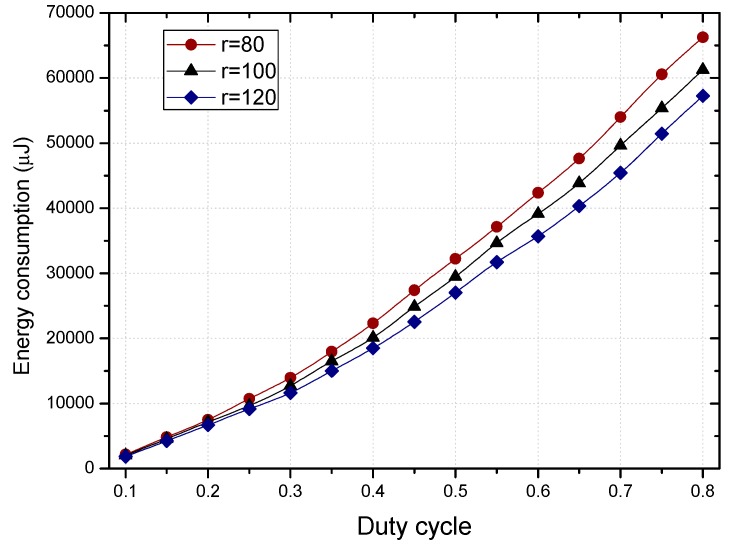
The energy consumption increases as the duty cycle increases.

**Figure 7:**
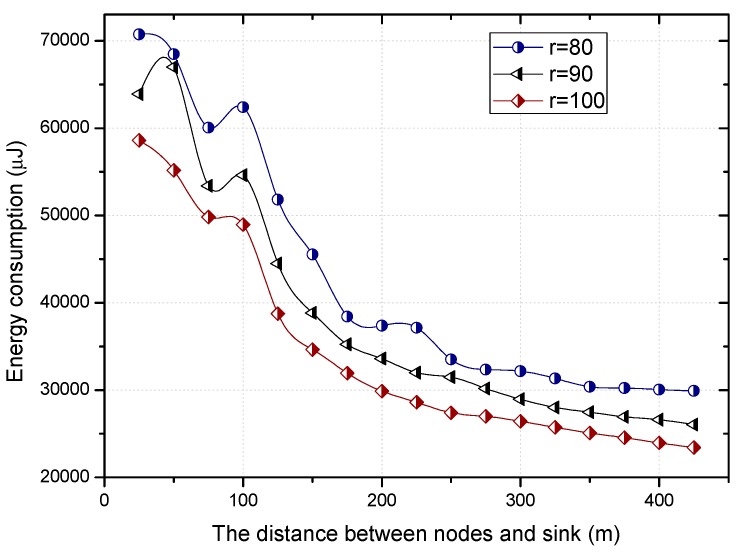
The energy consumption in different regions of the network.

As shown in Figure 6, the larger the duty cycle is, the more energy the nodes consume. This is because the energy consumed by nodes in the active state is more than during sleep, and the larger the duty cycle is, the longer the time that the node in the active state is. Due to the imbalance of energy consumption in the network (see Figure 7), it is feasible to set up a part of the nodes with a duty cycle of 1 by using the residual energy of the non-hotspots.

**Figure 8:**
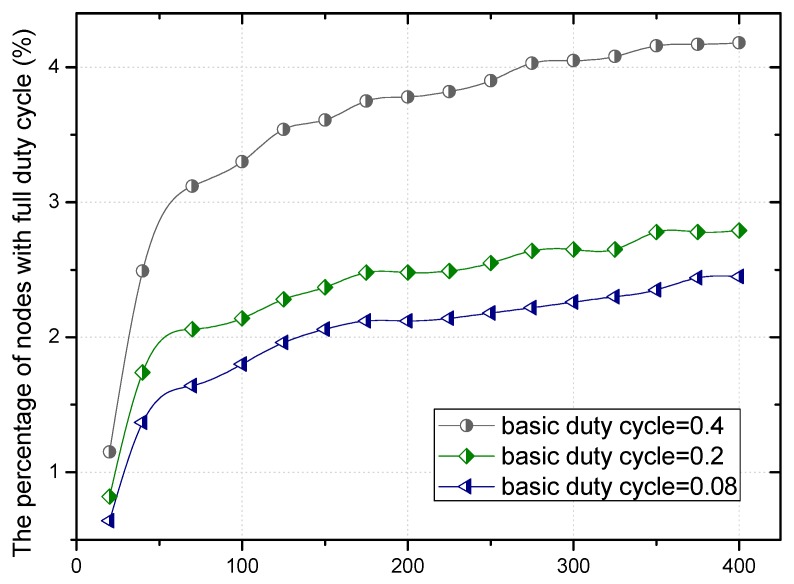
The proportion under different distance.

The residual energy in different regions can make the proportion of nodes with duty cycle of 1 different in the regions far or close to the sink, as can be seen in Figure 8.

**Figure 9:**
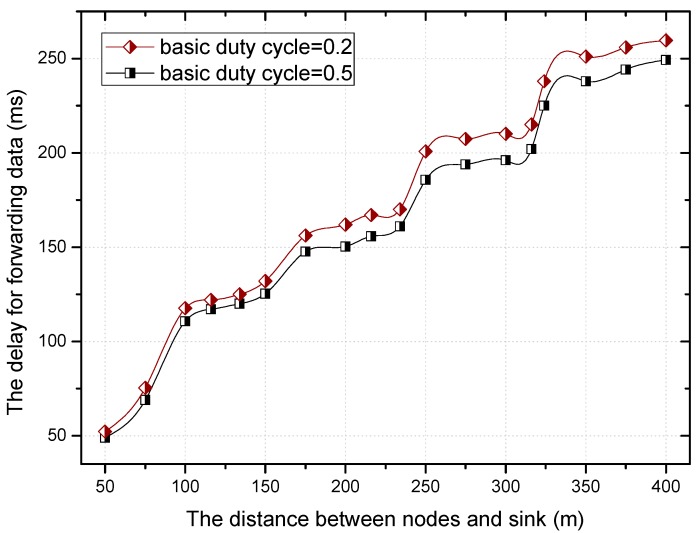
The delay for forwarding data under different distance.

The theoretical calculation (see corollary in Section 5.2) shows that the higher the proportion of nodes with a duty cycle of 1 is, the smaller the delay is, which can be seen in Figure 9. No matter the value of the base duty cycle, the delay for forwarding data increases with the increase of the distance to the sink. Combined with Figure 8 and Figure 9, we can see that the larger the base duty cycle is, the more the number of nodes with full duty cycle is, and the smaller the delay is.

**Figure 10:**
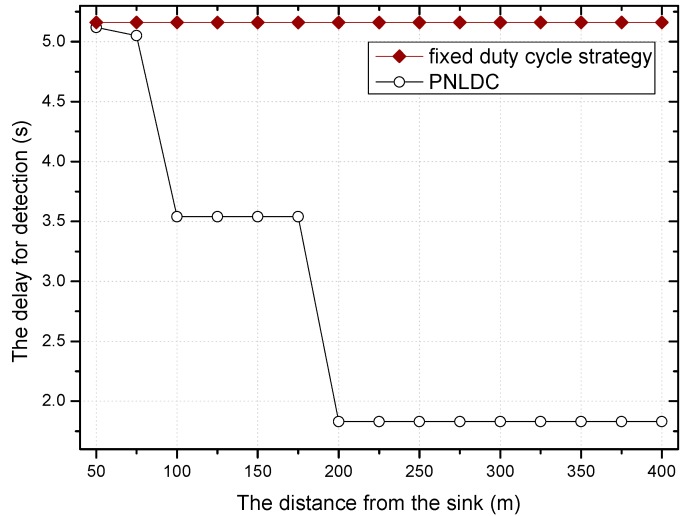
The delay for detection under different distance.

**Figure 11:**
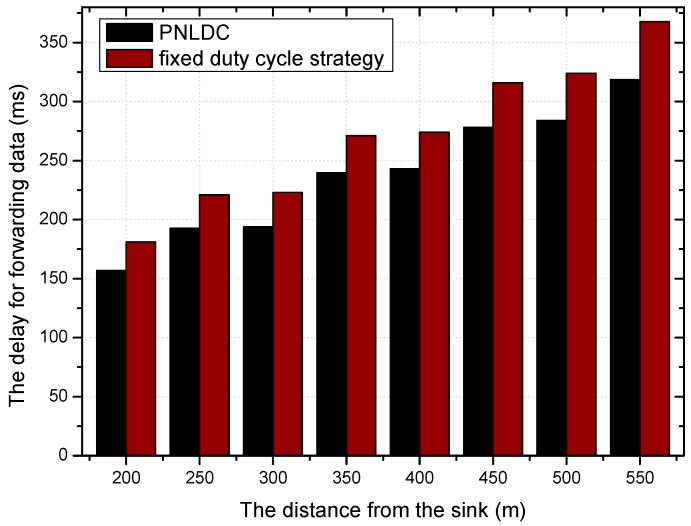
The delay for forwarding data under different distance.

On the basis of the above analysis, this paper proposes a better approach called the PNLDC approach, which can effectively reduce the delay. Figure 10 and Figure 11 compare the different performances of the fixed duty cycle strategy and the PNLDC approach in two aspects, delay for detection and delay for forwarding, respectively. It can be observed that the PNLDC approach performs better than the fixed duty cycle strategy in reducing delay.

### 4.2. The PNLDC Approach Design

The core thought of the PNLDC approach is: take full advantage of the residual energy in non-hotspots while not affecting the network lifetime, and reduce the delay by selecting partial nodes with a duty cycle of 1.

**Figure 12:**
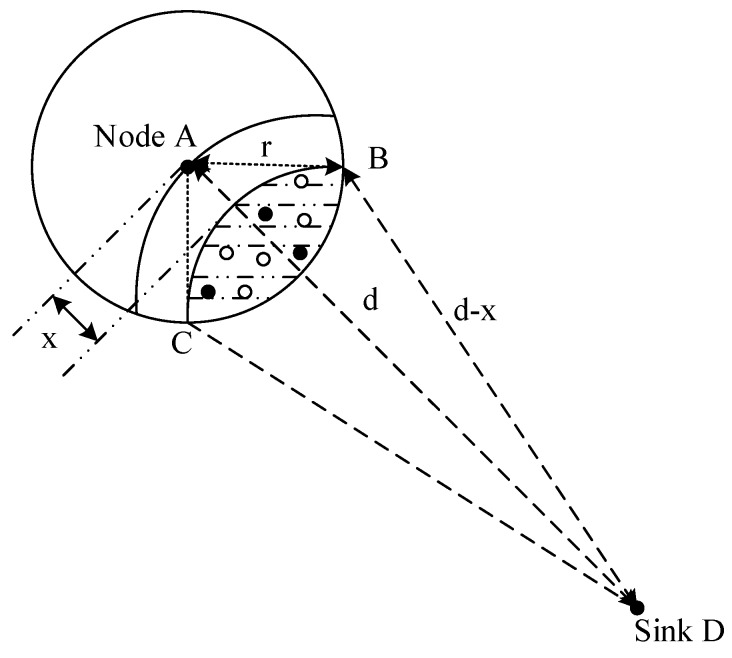
The range of candidate forwarding nodes.

The forwarding nodes set of nodes is first defined here. The range of the forwarding nodes set is formed by intersecting the circular transmission range of the sender with an arc that is parallel to the arc passing through the center of the circle. Suppose the parallel distance between these two arcs is *x*, and the transmission range of the sender is *r*. As shown in Figure 12, nodes in the shadow region are the candidate forwarding nodes. The distance between the sink and candidate forwarding nodes is closer to the distance from the sender node to the sink, so as to avoid selecting forwarders that are far from the sink.

Because the node density in the network is ρ, the number of the candidate forwarding nodes (denoted as α) can be calculated by Equation (8).

(8) $$\alpha =\rho \cdot \left[{\left(d-x\right)}^{2}\cdot \left(\cos^{-1}\frac{d^{2}+{\left(d-x\right)}^{2}-r^{2}}{2d\cdot \left(d-x\right)}\right)+\left(\cos^{-1}\frac{r^{2}+d^{2}-{\left(d-x\right)}^{2}}{2dr}\right)\cdot r^{2}-\sin\left(\cos^{-1}\frac{r^{2}+d^{2}-{\left(d-x\right)}^{2}}{2dr}\right)\cdot rd\right]$$

where the area of the range of the forwarding nodes set is calculated by the area of fan-shaped DBC plus the area of fan-shaped ABC minus the area of quadrilateral ABDC.

Next, to the phase that is the focus of this paper. According to Theorem 1, the greater the distance between the sender node and sink, the more the remaining energy. Thus, the duty cycle of partial nodes in the non-hot area is set to 1 based on the remaining energy in this region. And the higher the proportion of the selected nodes with full duty cycle in the area farther away from the sink.

**Theorem** **1.** *For the nodes with a distance of x from the sink, the distance between its forwarding nodes and the sink is $x^{\prime }$, then the remaining energy can be calculated by:*

(9) $$E_{REM}^{x}=\left(\varepsilon_{R}^{\prime }-\varepsilon_{R}^{x}\right)\cdot \xi_{R}^{x}+\left(\varepsilon_{T}^{\prime }-\varepsilon_{T}^{x}\right)\cdot \xi_{T}^{x}+\left(\varsigma_{R}^{x}+\varsigma_{T}^{x}-\varsigma_{R}^{\prime }-\varsigma_{T}^{\prime }\right)\cdot T_{COM}$$

**Proof.**  The remaining energy is related to the transmission of the packets, the reception of the packets, the low power listening, and the sensing duration. According to [65], we can get: $E_{REM}^{x}=E_{T}^{\prime }+E_{R}^{\prime }+E_{SEN}^{\prime }+E_{LPL}^{\prime }-E_{T}^{x}-E_{R}^{x}-E_{SEN}^{x}-E_{LPL}^{x}=\left(\varepsilon_{R}^{\prime }-\varepsilon_{R}^{x}\right)\cdot \xi_{R}^{x}+\left(\varepsilon_{T}^{\prime }-\varepsilon_{T}^{x}\right)\cdot \xi_{T}^{x}+\left(\varsigma_{R}^{x}+\varsigma_{T}^{x}-\varsigma_{R}^{\prime }-\varsigma_{T}^{\prime }\right)\cdot T_{COM}$, where $\varepsilon_{T}^{x}$ denotes the number of packets sent by the node *x* away from the sink and $\varepsilon_{R}^{x}$ is the number of packets received by the node *x* away from the sink. The calculation of $\varepsilon_{T}^{x}$ and $\varepsilon_{R}^{x}$ will be given in the Section 5.1. □

At the stage of optimizing the duty cycle, the PNLDC approach selects partial nodes and sets their duty cycle to 1, so as to take full advantage of the residual energy and reduce the delay. The selection of nodes with full duty cycle in different regions is based on Theorem 2.

**Theorem** **2.** *For the region x away from the sink, the proportion of nodes with full duty cycle (denoted as θ) can be calculated by the following equation.*

(10) $$\begin{array}{c} \theta =\left(\varepsilon_{R}^{x}-\varepsilon_{R}^{\prime }\right)\cdot \xi_{R}/\partial \\ \partial =\left(\mu_{R}-\mu_{S}\right)\left(\delta_{COM}-1\right)T_{COM}+\left(\mu_{SEN}-\mu_{S}\right)\left(\delta_{SEN}-1\right)T_{SEN}+\left(\varsigma_{R}^{x}+\varsigma_{T}^{x}-\varsigma_{R}^{\prime }-\varsigma_{T}^{\prime }\right)\cdot T_{COM}+\xi_{T}^{\prime }\varepsilon_{T}^{\prime }-\xi_{T}^{x}\varepsilon_{T}^{x} \end{array}$$

**Proof.**  Before the adjustment of the duty cycle of partial nodes in this phase, all nodes in the region *x* away from the sink have the same duty cycle. As mentioned earlier in this paper, it takes a lot of energy to change the nodes from the original duty cycle to the full duty cycle (that is, the duty cycle is 1). Considering that the regions far away from the sink have available remaining energy, this part of energy can be used to complete the operation in this phase. After the selection of the nodes with full duty cycle and the optimization of their duty cycle, the energy consumption of the nodes in the region *x* away from the sink will be equal to the energy consumption of nodes very close to the sink, which can be expressed by the formula:

(11) $$\left(E_{T}^{\prime }+E_{SEN}^{\prime }+E_{LPL}^{\prime }-E_{T}^{x}-E_{SEN}^{x}-E_{LPL}^{x}\right)\theta =E_{R}^{x}-E_{R}^{\prime }$$

The above formula can also be expanded as follows:

(12) $$\left[\left(\mu_{R}-\mu_{S}\right)\left(\delta_{COM}^{\prime }-\delta_{COM}^{x}\right)T_{COM}+\left(\mu_{SEN}-\mu_{S}\right)\left(\delta_{SEN}^{\prime }-\delta_{SEN}^{x}\right)T_{SEN}+\left(\varsigma_{R}^{x}+\varsigma_{T}^{x}-\varsigma_{R}^{\prime }-\varsigma_{T}^{\prime }\right)T_{COM}+\xi_{T}^{\prime }\varepsilon_{T}^{\prime }-\xi_{T}^{x}\varepsilon_{T}^{x}\right]\theta =\left(\varepsilon_{R}^{x}-\varepsilon_{R}^{\prime }\right)\xi_{R}$$

Because the duty cycle of nodes with full duty cycle is 1, then

(13) $$\left[\left(\mu_{R}-\mu_{S}\right)\left(\delta_{COM}-1\right)T_{COM}+\left(\mu_{SEN}-\mu_{S}\right)\left(\delta_{SEN}-1\right)T_{SEN}+\left(\varsigma_{R}^{x}+\varsigma_{T}^{x}-\varsigma_{R}^{\prime }-\varsigma_{T}^{\prime }\right)T_{COM}+\xi_{T}^{\prime }\varepsilon_{T}^{\prime }-\xi_{T}^{x}\varepsilon_{T}^{x}\right]\theta =\left(\varepsilon_{R}^{x}-\varepsilon_{R}^{\prime }\right)\xi_{R}$$

Based on the Formula (13), the proportion of nodes with full duty cycle can be calculated by the following equation.

(14) $$\theta =\left(\varepsilon_{R}^{x}-\varepsilon_{R}^{\prime }\right)\cdot \xi_{R}/\partial$$

(15) $$\partial =\left(\mu_{R}-\mu_{S}\right)\left(\delta_{COM}-1\right)T_{COM}+\left(\mu_{SEN}-\mu_{S}\right)\left(\delta_{SEN}-1\right)T_{SEN}+\left(\varsigma_{R}^{x}+\varsigma_{T}^{x}-\varsigma_{R}^{\prime }-\varsigma_{T}^{\prime }\right)\cdot T_{COM}+\xi_{T}^{\prime }\varepsilon_{T}^{\prime }-\xi_{T}^{x}\varepsilon_{T}^{x}$$

□

**Figure 13:**
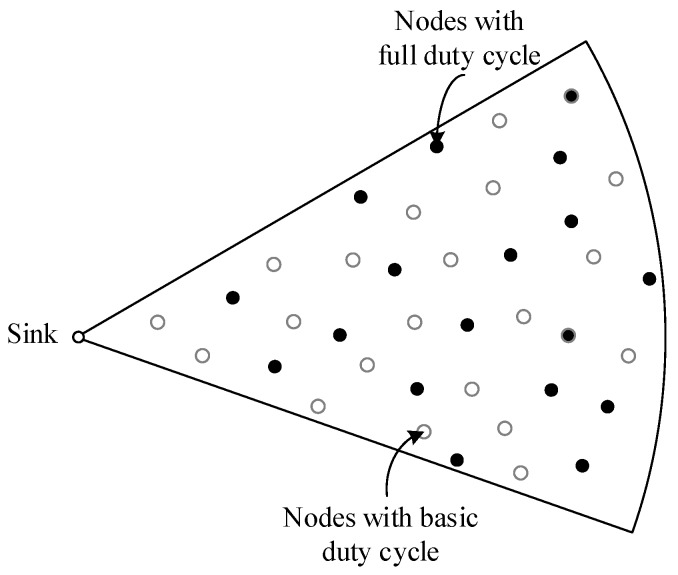
The distribution of two kinds of nodes relative to the sink.

According to the above theorems, the greater the distance between the area and the sink, the higher the proportion of the selected nodes with full duty cycle in this area, which can be seen from Figure 13.

**Figure 14:**
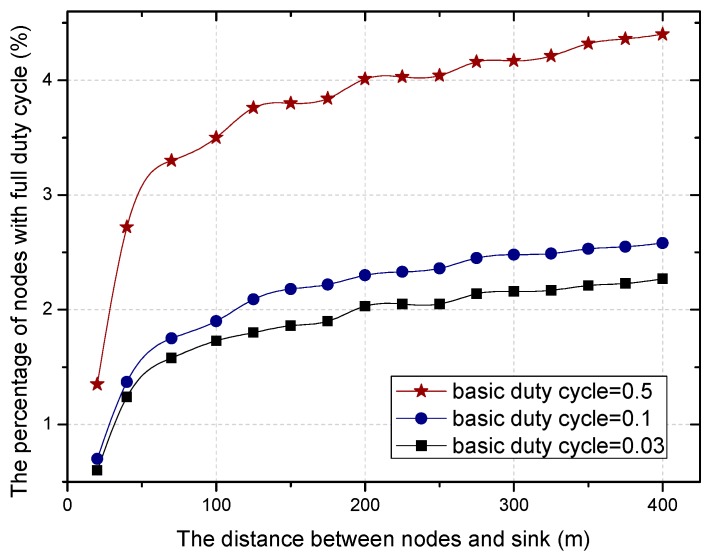
The proportion under different distance.

Based on Equation (15), the relationship between the proportion of nodes with full duty cycle and the distance to the sink is more intuitively shown in Figure 14. The proportion of nodes with full duty cycle increases with the increase of distance. This is because the residual energy is much more when the distance between the node and the sink is larger. Combined with the changing trend of the proportion under a different duty cycle, the larger the basic duty cycle is, the greater the number of nodes with a full duty cycle.

## 5. Performance Analysis and Simulation Results

### 5.1. Energy Consumption and Network Lifetime

In the PNLDC approach, nodes mainly have four different states: low power listening, sensing, sending packets, and receiving packets. Because in the network the amount of data packets is large and the reception and transmission of packets are the main energy-consuming operations, the energy consumption is serious. The energy consumption of the node *x* away from the sink can be calculated by the following equation.

(16) $$\xi_{TOT}^{x}=\xi_{T}^{x}\varepsilon_{T}^{x}+\xi_{R}^{x}\varepsilon_{R}^{x}+\xi_{LPL}^{x}+\xi_{SEN}^{x}$$

where $\xi_{TOT}^{x}$ represents the total energy consumption of the node, $\xi_{T}^{x}$ and $\xi_{R}^{x}$ are the energy consumed in the transmission and reception respectively, and $\xi_{LPL}^{x}$ is the energy consumption of low power listening and $\xi_{SEN}^{x}$ represents the energy consumption in sensing. $\varepsilon_{T}^{x}$ is the number of packets in transmission and $\varepsilon_{R}^{x}$ represents the number of packets in reception. According to Ref. [61], assuming that the distance between the node and sink is *x*, *N* packets are sent by this node, then

(17) $$N=\frac{\left(\tau +1\right)\cdot \tau r}{2x}+\tau +1$$

where τ is an integer so that τr+x is less than the radius of this network.

When the probability of event production is β, the amount of packets in reception is

(18) $$\varepsilon_{T}^{x}=\left(\frac{\left(\tau +1\right)\cdot \tau r}{2x}+\tau +1\right)\beta$$

The amount of packets sent by the node is equal to the amount of packets received by it plus the packets generated by itself.

(19) $$\varepsilon_{R}^{x}=\varepsilon_{T}^{x}+\beta$$

For the nodes with a distance of *x* from the sink, the energy consumed in the transmission can be presented as the following equation.

(20) $$\xi_{T}^{x}=\left(T_{B}\mu_{T}+T_{ACK}\mu_{R}\right)\frac{T_{COM}\left(1-\delta_{COM}\right)}{2\left(T_{B}+T_{ACK}\right)}+T_{P}\mu_{T}$$

where $T_{B}\mu_{T}$ denotes the energy consumption in the preamble duration, $T_{ACK}\mu_{R}$ represents the energy consumed by the transmission of ACK message and $T_{P}\mu_{T}$ represents the energy consumption of the packet transmission.

The energy consumption in the reception of a packet can be expressed as:

(21) $$\xi_{R}^{x}=T_{B}\mu_{R}+T_{ACK}\mu_{T}+T_{P}\mu_{R}$$

The energy consumption of sensing can be expressed by:

(22) $$\xi_{SEN}^{x}=\left(1-\delta_{SEN}\right)\mu_{S}+\mu_{SEN}\delta_{SEN}$$

where $\delta_{SEN}$ is the sensing duty cycle, $\mu_{SEN}$ is the power consumption of sensing when nodes are in the active state and $\mu_{S}$ is the power used when nodes are in the sleep state.

For the nodes *x* away from the sink, the energy consumption of low power listening can be calculated as:

(23) $$\xi_{LPL}^{x}=\delta_{COM}\mu_{R}+\left(1-\delta_{COM}\right)\mu_{S}-\varsigma_{T}^{x}-\varsigma_{R}^{x}$$

where the first part of Equation (24) represents the energy used by listening and the second part represents the energy consumption of sleeping. According to Ref. [61], $\varsigma_{T}^{x}$ and $\varsigma_{R}^{x}$ can be calculated by the following equations.

(24) $$\varsigma_{T}^{x}=\frac{\varepsilon_{T}^{x}}{T_{COM}}\left\{T_{B}\mu_{R}+\left[T_{B}+T_{ACK}+\frac{T_{COM}\left(1-\delta_{COM}\right)}{2}\right]\mu_{S}\right\}$$

(25) $$\varsigma_{T}^{x}=\frac{\varepsilon_{R}^{x}}{T_{COM}}\left(T_{B}\mu_{R}+\left(T_{P}+T_{ACK}\right)\mu_{S}\right)$$

**Figure 15:**
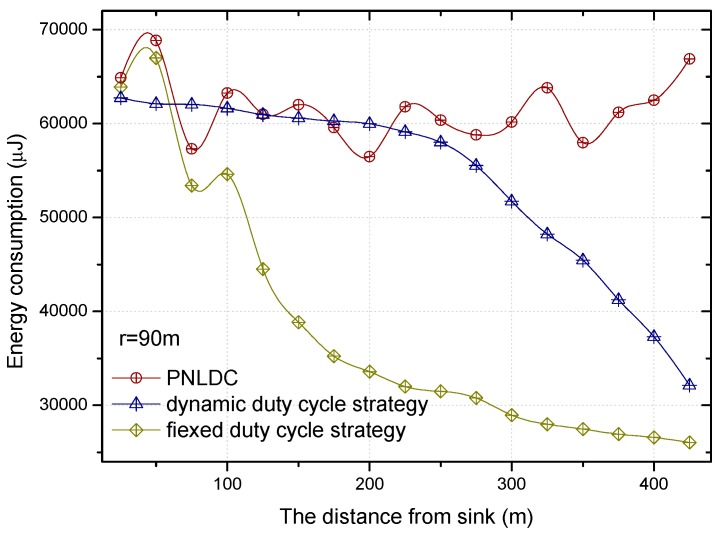
The energy consumption under different distance.

**Figure 16:**
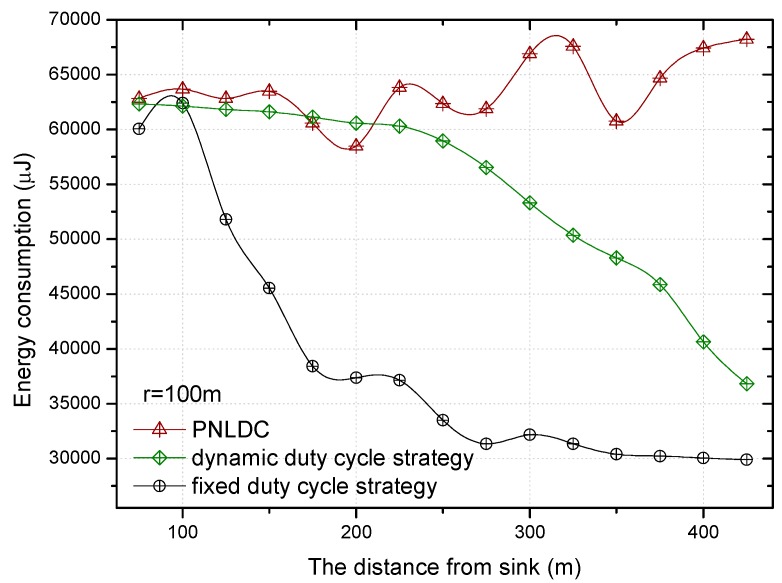
The energy consumption under different distance.

The comparison of energy consumption of the fixed duty cycle strategy, dynamic duty cycle strategy, and PNLDC approach is shown in Figure 15 and Figure 16, when the basic duty cycle is 0.2. It can be obtained from the change curve of energy consumption in various regions that energy consumption decreases with the increase of distance to the sink in the fixed duty cycle strategy. Unlike the fixed duty cycle strategy, although the energy consumption in different regions has a certain fluctuation, it is maintained within a certain range. Therefore, compared with the fixed duty cycle strategy, the PNLDC approach can make use of network energy more effectively. And the energy consumption curves of the dynamic duty cycle strategy are different from those of the other two strategies. For the sensor nodes in the hotspots, the energy consumption of the dynamic duty cycle strategy is only slightly lower than that of the PNLDC approach. For the nodes in the non-hotspots, the energy utilization of the dynamic duty cycle strategy is obviously lower than that of the PNLDC approach. This is because the duty cycle in the dynamic duty cycle strategy varies according to the traffic load in the network [50], that is, the duty cycle is large when sensors have high traffic loading. Therefore, in the non-hotspots far away from the sink, the duty cycle in the dynamic duty cycle strategy is relatively small. In contrast, in the PNLDC approach, partial nodes in the region far away from the sink are selected as the nodes with a full duty cycle. Thus, for the region far away from the sink, the PNLDC approach can make better use of energy than the dynamic duty cycle strategy.

The network lifetime is defined as the time that the first node in the network dies out due to energy exhaustion, which is closely related to the initial energy and the maximum of energy consumption. Suppose that the initial energy in the network is $E_{ini}$, the network lifetime is a result of the initial energy divided by the average energy consumed in a cycle, which can be expressed as follows:

(26) $$L=\frac{E_{ini}}{max\left(E_{T}^{x}+E_{R}^{x}+E_{LPL}^{x}+E_{SEN}^{x}\right)}$$

where $E_{T}^{x}=\varepsilon_{T}^{x}\xi_{T}^{x}$, $E_{R}^{x}=\varepsilon_{R}^{x}\xi_{R}^{x}$, $E_{SEN}^{x}=T_{SEN}\xi_{SEN}^{x}+\mu_{S}$, and $E_{LPL}^{x}=T_{COM}\left[\delta_{COM}\mu_{R}-\frac{\left(1-\delta_{COM}\right)\mu_{S}}{2}-\varsigma_{T}^{x}\right]-\left(T_{B}+T_{ACK}\right)\mu_{S}-T_{B}\mu_{R}$.

**Figure 17:**
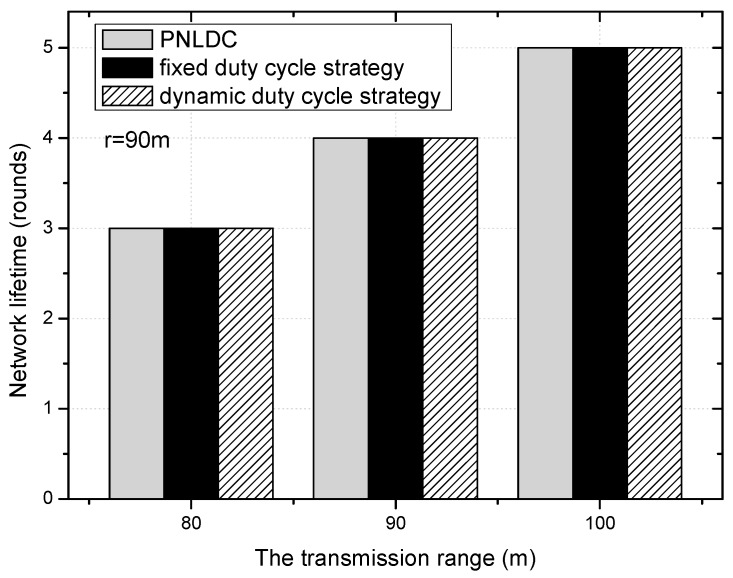
The network lifetime under different transmission range.

Figure 17 shows the network lifetime of the PNLDC approach, the dynamic duty cycle strategy, and the fixed duty cycle strategy under three values of transmission range. As can be seen in the figure, the network lifetime increases with the increase of the transmission range. Compared with the fixed duty cycle strategy and dynamic duty cycle strategy, the network lifetime is not reduced in the PNLDC approach. This is because the energy consumed by nodes with the duty cycle of 1 is derived from the residual energy of nodes in the network. In combination with the above analysis, the PNLDC approach can increase the utilization of energy without reducing the network lifetime.

### 5.2. Delay of Forwarding Data

In the process of data transmission, besides sending and receiving data, there are also the sending and receiving of the preamble and the ACK. We analyze the delay for forwarding data in this section.

**Theorem** **3.** *In the PNLDC approach, if the sender node is x away from the sink and the proportion of nodes with full duty cycle is θ, then the average delay for forwarding data per hop can be calculated by Equation (27).*

(27) $$Y_{hop}=\left[T_{P}+T_{B}+T_{ACK}+\frac{T_{COM}{\left(1-\delta_{COM}\right)}^{2}}{2}\right]\left(1-\theta asin\frac{R_{S}}{x}\right)$$

*where $\delta_{COM}$ is the communication duty cycle, $T_{COM}$ represents the communication duration, and $T_{P}$, $T_{B}$, $T_{ACK}$ represent the duration of packet, preamble and ACK message, respectively.*

**Proof.**  Considering that the state of the receiver node, the probability that this receiver is in the active state when the sender node starts sending a packet is $\delta_{COM}$. In such a situation, the transmission delay is $T_{P}+T_{B}+T_{ACK}$. In contrast to this case, the receiver is in the sleep state, the corresponding probability is $1-\delta_{COM}$. To calculate the average transmission delay for each hop, the best and worst situations needs to be taken into account. In the best situation, the sender node starts sending a packet when the corresponding receiver starts the low power listening. Therefore, the packet will be successfully transferred to the receiver after $T_{P}+T_{B}+T_{ACK}$. In the worst situation, the sender node needs to wait for the whole duration of sleep state. Because the receiver should send the ACK back to the sender after receiving the entire preamble, this may require two transmissions of preambles before the packet transmission in the worst case situation. Thus, the transmission delay is $T_{P}+T_{B}+T_{ACK}+T_{COM}\left(1-\delta_{COM}\right)$. Based on the above analysis, according to [65], the average delay per hop in the transmission path can be expressed as:

(28) $$Y_{basic}=T_{P}+T_{B}+T_{ACK}+\frac{T_{COM}{\left(1-\delta_{COM}\right)}^{2}}{2}$$

This equation is suitable for the case that all nodes take the same duty cycle.

In the PNLDC approach, because some nodes are selected as nodes with full duty cycle, if the packet meets these nodes on the transmission path, the delay after detection will be decreased. According to [66], the probability of nodes with a full duty cycle on the transmission path is $\theta a\sin\frac{R_{S}}{x}$. Thus, the delay for forwarding data per hop can be calculated by Equation (28). □

The proportion of nodes with a full duty cycle in the region *x* away from the sink is calculated by Equations (14) and (15) in Section 4.2.

**Corollary** **1.** *The total delay for forwarding data can be calculated as:*

(29) $$Y_{x,hop}=\vartheta \left[T_{P}+T_{B}+T_{ACK}+\frac{T_{COM}{\left(1-\delta_{COM}\right)}^{2}}{2}\right]\left(1-\theta asin\frac{R_{S}}{x}\right)$$

*where is $\vartheta =\lceil \frac{x}{r}\rceil +1$ and 0<x≤R.*

**Proof.**  Assuming that the sender node is *x* away from the sink, and the packet sent by it is required to pass through ϑ hops, this means that there are ϑ nodes on the transmission path of a packet. Therefore, the total delay for forwarding data can be expressed as the product of the delay of each hop and the number of hops. □

**Figure 18:**
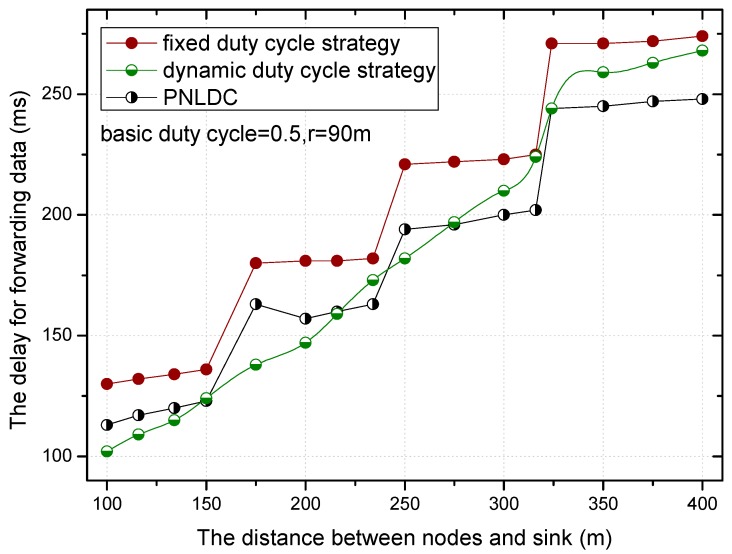
The delay for forwarding data under different distance.

**Figure 19:**
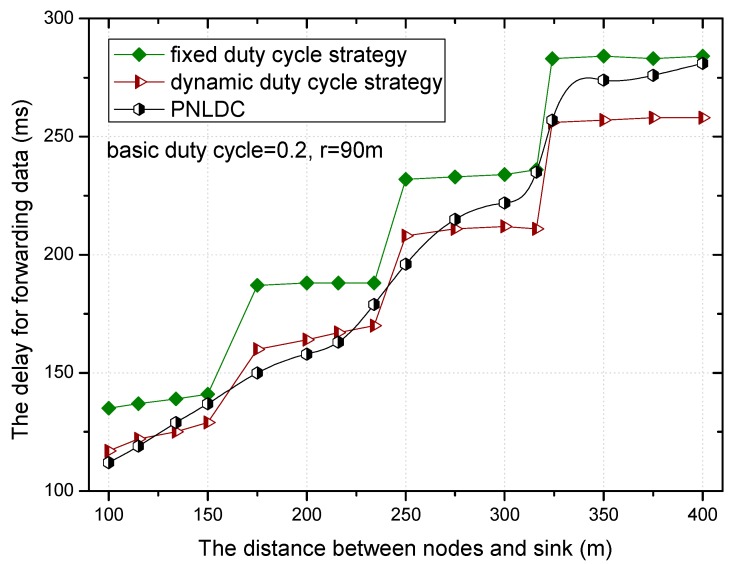
The delay for forwarding data under different distance.

Figure 18 and Figure 19 show the relationship between the delay for forwarding data and the distance between nodes and the sink. At the same time, the performance of the delay for forwarding data of the fixed duty cycle strategy, the dynamic duty cycle strategy and PNLDC approach are compared. With the increase of the distance between the node and sink, the total delay for forwarding data also increases, which is obvious. It can also be obtained that the greater the duty cycle, the smaller the delay for forwarding data. This is because the greater the duty cycle, the more time the node is in the active state, that is, the shorter the time that sender needs to wait. Compared with the fixed duty cycle strategy, the delay for forwarding data is reduced by 8.9~26.4% in the PNLDC approach. Therefore, in terms of delay for forwarding data, the PNLDC approach performs better than the fixed duty cycle strategy. And compared with the dynamic duty cycle strategy, the PNLDC approach has the advantage that the delay for forwarding data is smaller in the region far away from the sink. This is because in the non-hotspots far away from the sink, the duty cycle of the nodes in the dynamic duty cycle strategy is smaller than that in the PNLDC approach.

### 5.3. Delay for Detection

In this section, we analyze the delay for detection in the PNLDC approach. According to Ref. [65], for a sensor node with a distance of *x* from the sink, the limitation on the probability of a missed packet (denoted as $P_{mis}$) can be expressed by the following formula.

(30) $$\begin{array}{c} P_{mis}\geq {\left\{1-\frac{R_{S}}{R}\left[\left(1-\delta_{SEN}\right)P\left\{A\right\}+\delta_{SEN}\right]\right\}}^{\alpha }\\ \mathrm{where}\;P\left\{A\right\}=\{\begin{array}{c} \frac{4R_{S}}{\pi \gamma T_{SEN}\left(1-\delta_{SEN}\right)},\;if\;2R_{S}/\gamma <T_{SEN}\left(1-\delta_{SEN}\right)\\ \frac{4R_{S}-2\sqrt{4{R_{S}}^{2}-{\left(1-\delta_{SEN}\right)}^{2}\gamma^{2}}}{\pi \gamma T_{SEN}\left(1-\delta_{SEN}\right)}+1-\frac{2a\sin\left(\frac{\gamma \left(1-\delta_{SEN}\right)}{2R_{S}}\right)}{\pi },\;else \end{array} \end{array}$$

where the radius of the network is *R*, the sensing range of the node is $R_{S}$, α is the number of nodes in this range, γ is the speed of the packet and θ is the proportion of nodes with full duty cycle.

Equation (31) is based on the network that adopts the same duty cycle. In the PNLDC approach, the probability of a missed packet ($P_{mis}$) will alter because the duty cycle of some nodes is set to 1. The change of $P_{mis}$ is related to the proportion of nodes with a full duty cycle. Therefore, the formula applicable to this paper is as follows.

(31) $$P_{mis}\geq C_{\alpha }^{\theta \alpha }{\left(\frac{R_{S}}{R}\right)}^{2\theta \alpha }{\left\{1-\frac{R_{S}}{R}\left[\left(1-\delta_{SEN}\right)P\left\{A\right\}+\delta_{SEN}\right]\right\}}^{\alpha -\theta \alpha }$$

Based on the above analysis, next considering the case of detecting multiple packets at the same time.

First we analyze the probability that all packets are not detected (denoted as $P_{misa}$). It can be calculated by:

(32) $$P_{misa}={\left(P_{mis}\right)}^{\alpha_{m}}$$

where $\alpha_{m}$ represents the number of packets that are sent to the node at the same time, and $P_{mis}$ is the probability of a missed packet in the case of only a packet entering the range of perception. So, $1-P_{misa}$ represents the probability that at least one packet of $\alpha_{m}$ packets is detected.

The probability that at least one packet is not detected (denoted as $P_{misp}$) can be derived:

(33) $$P_{misp}=1-{\left(1-P_{mis}\right)}^{\alpha_{m}}$$

**Figure 20:**
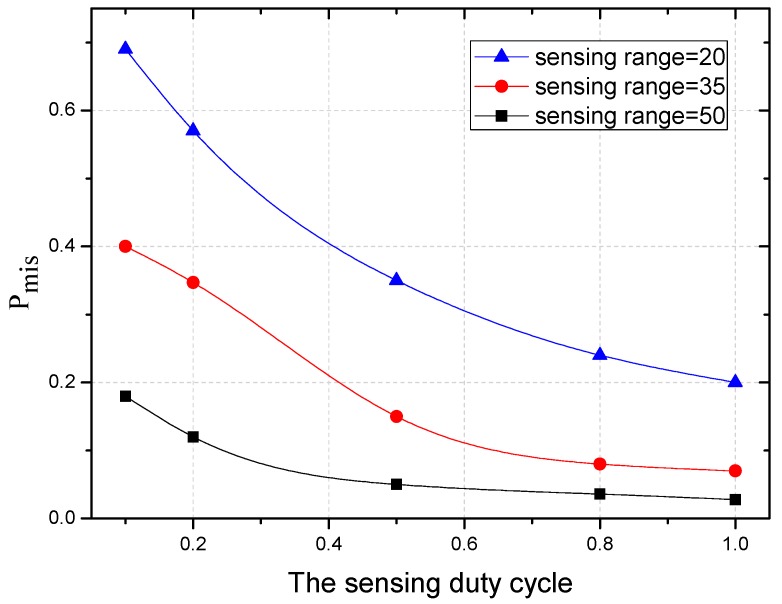
The $P_{mis}$ under different sensing duty cycle.

Figure 20 shows the variation trend of $P_{mis}$ under the different sensing duty cycle. With the decrease of the duty cycle, $P_{mis}$ also decreases, which coincides with Equation (31). And with the narrowing of the sensing range, the probability of missed a packet ($P_{mis}$) increases.

**Figure 21:**
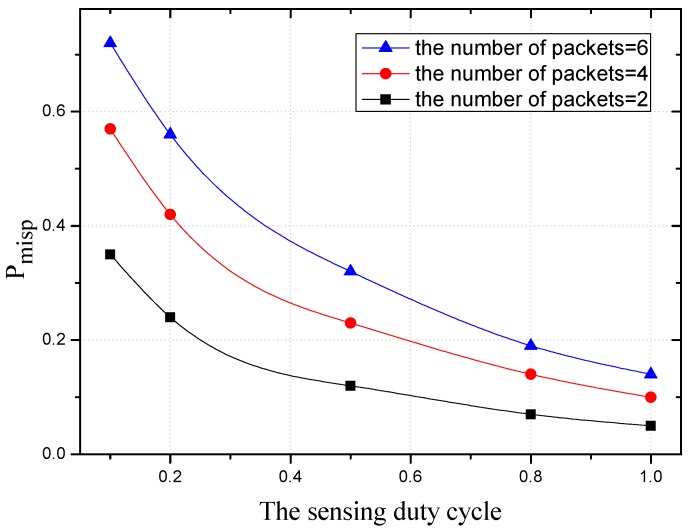
The $P_{misp}$ under different duty cycle.

The variation trend of $P_{misp}$ under the different sensing duty cycle is shown in Figure 21. Here, three values of the number of packets sent to the node (2, 4 and 6) are considered. It can be obtained from the figure: as the number of packets increases, $P_{misp}$ also increases. In the same way as Figure 20, $P_{misp}$ decreases as the sensing duty cycle increases, which is consistent with Equations (31) and (33).

In the PNLDC approach, assuming that the distance between the sensor node and the sink is *x*. The probability of missed packets can be calculated using the infinitesimal method [65]. A fan-shaped region with a width of *dx* and an angle of dφ is selected. Thus the area of this region is xdxdφ. Then, the weighted probability that at least one packet is not detected and the weighted probability that all packets are not detected can be calculated by the following equations.

(34) $$P_{misp}^{x}=\int_{0}^{R}\int_{0}^{2\pi }x\cdot P_{misp}dxd\varphi$$

(35) $$P_{misa}^{x}=\int_{0}^{R}\int_{0}^{2\pi }x\cdot P_{misa}dxd\varphi$$

The probability of missed packets will affect the delay for detection. This delay can be measured by the time that the packet is first detected by the sensor node after entering the perception region of nodes.

According to [65], the delay for detection can be calculated as:

(36) $$Y_{DET}=\{\begin{array}{c} C_{\alpha }^{\theta \alpha }{\left(\frac{R_{S}}{R}\right)}^{2\theta \alpha }\frac{{\left(\pi R^{2}\right)}^{\alpha -\theta \alpha +1}-{\left(2\psi_{m}R_{S}-\pi R^{2}\right)}^{\alpha -\theta \alpha +1}}{2R_{S}\cdot \gamma \cdot {\left(\pi R^{2}\right)}^{\alpha -\theta \alpha }\cdot \left(\alpha -\theta \alpha +1\right)},\;if\;\frac{d}{\gamma }>h\\ \frac{\int_{0}^{\psi_{m}}\left[C_{\alpha }^{\theta \alpha }{\left(\frac{R_{S}}{R}\right)}^{2\theta \alpha }{\left(1-\frac{2\psi R_{S}}{\pi R^{2}}\right)}^{\alpha -\theta \alpha }+\sum_{i=1}^{\alpha }P\left\{B\right\}\right]d\psi }{\gamma },\;else \end{array}$$

where *d* denotes the length of the intersection between the packet transmission path and the sensing region of the sensor node, γ is the speed of the packet transmission and $h=T_{SEN}\left(1-\delta_{SEN}\right)$. ψ is the length of the packet transmission path in the sensing region, and $\psi_{m}$ is the average maximum value of ψ. And according to [65], $P\left\{B\right\}=\left(\begin{array}{c} \alpha \\ i \end{array}\right){\left(1-\frac{2\psi R_{S}}{\pi R^{2}}\right)}^{\alpha -i}{\left(P\left\{C\right\}\cdot \frac{2\psi R_{S}}{\pi R^{2}}\right)}^{i}$, where P{C} represents the probability of the path of a packet passes through a specific perception region of a sensor node that does not detect the packet, and *i* is the number of this kind of nodes. If $d/\gamma >T_{SEN}\left(1-\delta_{SEN}\right)$, P{C}=0. Else if $d/\gamma \leq T_{SEN}\left(1-\delta_{SEN}\right)$, $P\left\{C\right\}=\frac{R_{S}}{R}\left(1+P\left\{A\right\}\right)\left(1-\delta_{SEN}\right)$.

Based on the above analysis, the weighted delay for detection can be calculated by Equation (37).

(37) $$Y_{DET}^{x}=\{\begin{array}{c} Y_{DET},\;if\;\frac{d}{\gamma }>T_{SEN}\left(1-\delta_{SEN}\right)\\ \int_{0}^{R}\int_{0}^{2\pi }x\cdot Y_{DET}dxd\varphi ,\;else \end{array}$$

where the sensor node is *x* away from the sink and the network radius is *R*.

**Figure 22:**
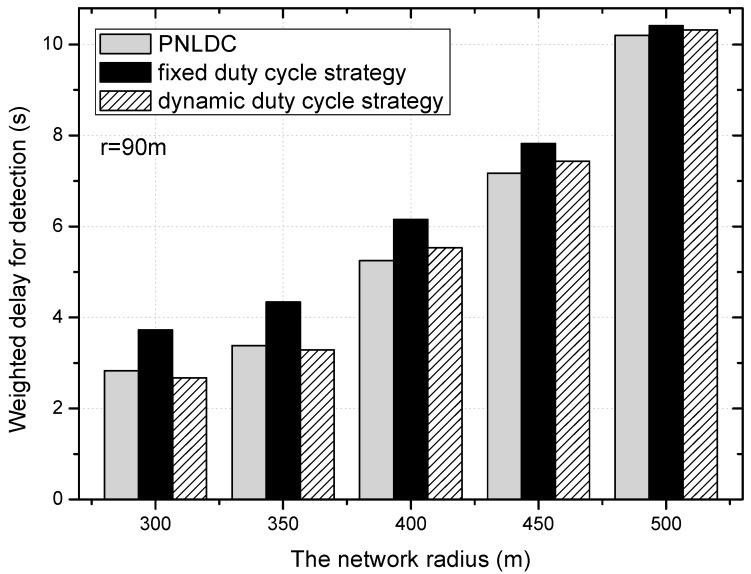
The delay for detection under different radius of network.

Figure 22 shows the variation trend of delay for detection under different radius of network, when the transmission range of the node is ninety meters and the basic duty cycle is 0.2. As can be seen in this figure, with the increase of the network radius, the delay for detection also increases. Compared with the dynamic duty cycle strategy, the overall performance of the PNLDC approach in terms of delay for detection is no worse than that of the dynamic duty cycle strategy. It can be obtained from the contrast between the grey and black columns that the PNLDC approach is actually better able to reduce the delay for detection. Compared with the fixed duty cycle strategy, the delay for detection is reduced by 2.1~24.6% in the PNLDC approach. Therefore, in terms of delay for detection, the PNLDC approach outperforms the fixed duty cycle strategy.

## 6. Conclusions and Future Work

In Industrial Wireless Sensor Networks, reducing delay is an important task. The previous strategies tended to reduce the delay by consuming a part of the energy and thus reduce the network lifetime. In this paper, a novel approach termed PNLDC is proposed. This approach is devoted to reducing the delay while ensuring that the lifetime is not less than the previous strategies. Compared with the strategies that all nodes adopt the fixed duty cycle, the PNLDC approach focuses on increasing the duty cycle of partial nodes in the network. PNLDC selects a certain proportion of nodes in the non-hotspots and sets their duty cycle to 1, which can reduce the transmission delay while using residual energy. So, the PNLDC approach can increase the utilization of energy and reduce delay without reducing the network lifetime, which can be obtained from the performance analysis and experimental results. At the same time, this approach has extensive applicability; it can be used not only to reduce the latency in IWSNs, but also to applications based on the Internet of things technology, which is included in our further studies.

## Figures and Tables

**Table 1:** System Parameters.

Parameter	Description	Value
$E_{ini}$	Initial energy	0.5 J
$T_{COM}$	Communication duration	100 ms
$\mu_{T}$	Power consumed by transmission	0.0511 W
$\mu_{R}$	Power consumed by reception	0.0588 W
$\mu_{S}$	Power consumed by sleeping	2.4 × 10^−7^ W
$T_{B}$	Preamble duration	0.26 ms
$T_{ACK}$	Acknowledge window duration	0.26 ms
$T_{P}$	Packet duration	0.93 ms
$T_{SEN}$	Sensing duration	15 s
